# Editorial: Transdiagnostic approach in studying mental health conditions: the contribution of fundamental and translational brain research for precise interventions

**DOI:** 10.3389/fnhum.2026.1854546

**Published:** 2026-05-07

**Authors:** Sandra Carvalho, Wolnei Caumo, Ana Rita Ferreira, Lia Fernandes, Jorge Leite

**Affiliations:** 1 Psychology Research Centre (CIPsi), Department of Basic Psychology, School of Psychology, University of Minho, Braga, Portugal; 2 Post-Graduate Program in Medical Sciences, School of Medicine, Universidade Federal do Rio Grande do Sul (UFRGS), Porto Alegre, Brazil; 3 Laboratory of Pain and Neuromodulation at Hospital de Clínicas de Porto Alegre (HCPA), Porto Alegre, Brazil; 4 Pain and Palliative Care Service at Hospital de Clínicas de Porto Alegre, Porto Alegre, Brazil; 5 Rede de Investigação em Saúde-Health, Department of Clinical Neurosciences and Mental Health, Faculty of Medicine, University of Porto, Porto, Portugal; 6 Psychiatry Service, Centro Hospitalar Universitário São João, Porto, Portugal; 7 RISE-Health (RISE-Health@UPT), Portucalense University, Porto, Portugal

**Keywords:** artificial intelligence, biomarkers, dimensional psychopathology, neurocognitive mechanisms, precision psychiatry, transdiagnostic, translational neuroscience

Over the past decades, mental health research and clinical practice have been largely organized around categorical diagnostic systems such as the Diagnostic and Statistical Manual of Mental Disorders (DSM) and the International Classification of Diseases (ICD). Although these systems have enhanced diagnostic consistency and communication in clinical practice, they also have notable drawbacks, such as substantial comorbidity among disorders, overlap of symptoms across diagnostic categories, variability within diagnoses, limited capacity to forecast treatment outcomes or the course of illness, and reduced cross-cultural reliability and validity (Cuthbert and Insel, 2013; Insel et al., 2010). These limitations have led to increasing recognition that mental disorders may be better understood in terms of underlying dimensions and mechanisms rather than discrete diagnostic categories or symptom clusters. Importantly, this shift is not only a change in classification but also reflects a move toward conceptualizing mental health and psychopathology as a set of dynamic, continuous, and interacting processes that unfold over time (Cuthbert and Insel, 2013; Insel et al., 2010). In this context, transdiagnostic and dimensional approaches have emerged as an important paradigm in psychopathology research, emphasizing shared psychological, cognitive, and neurobiological processes that cut across traditional diagnostic boundaries (Aldao et al., 2016; Dalgleish et al., 2020; Harvey et al., 2004). Frameworks such as the Research Domain Criteria (RDoC) have provided theoretical scaffolding and promoted multilevel approaches integrating behavioral, cognitive, physiological, and neural systems to better understand mental health and mental disorders (Insel et al., 2010; Insel, 2014). These approaches are closely linked to translational neuroscience and precision psychiatry, which aim to translate fundamental knowledge about brain and behavior into more precise and individualized interventions (Fernandes et al., 2017). Figure 1 provides an integrative schematic of this conceptual shift, illustrating how transdiagnostic symptom dimensions emerge from dynamic interactions between neurocognitive, biological, and environmental systems across time, and how these processes inform translational and precision psychiatry approaches.

**Figure 1:**
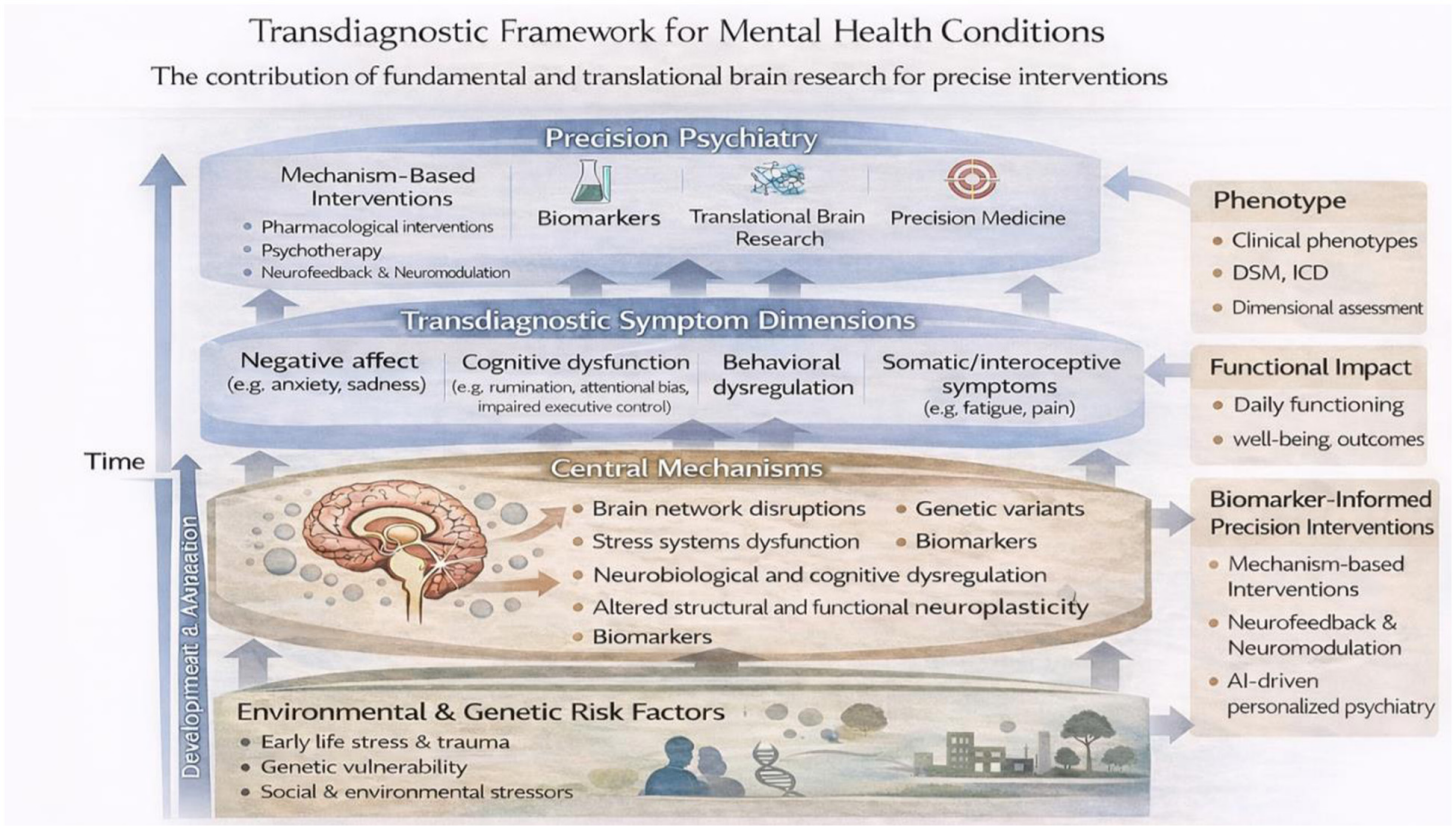
Multilevel transdiagnostic framework integrating dimensional psychopathology, neurocognitive and brain–body mechanisms, and biomarker-informed approaches within a translational and precision psychiatry perspective. The model illustrates the dynamic interactions between environmental and genetic risk factors, central mechanisms, and transdiagnostic symptom dimensions over time, supporting the development of mechanism-based and personalized interventions. The model is conceptually based on Carvalho and adapted for the purposes of this Research Topic.

Considering this background, this editorial brings together 11 articles that examine mental health from dimensional, transdiagnostic, neurocognitive, and translational perspectives. Although the studies address different populations, methodologies, and research questions, several common themes emerge across these contributions. These include dimensional approaches to psychopathology and assessment, transdiagnostic psychological processes, neurocognitive and brain–body mechanisms, mechanism-based interventions, and biomarker-informed approaches to mental health. These contributions reflect the ongoing transition in mental health research from disorder-based models toward dimensional, mechanism-based, and translational approaches that may support the development of more precise and personalized interventions.

Dimensional and transdiagnostic assessment approaches are also represented in this Research Topic. Macmillan et al. examined the latent structure of the DSM-5-TR Level 1 Cross-Cutting Symptom Measure in a large real-world psychiatric outpatient sample, identifying both general psychopathology and domain-specific symptom dimensions. Their findings support the use of dimensional and measurement-based approaches in clinical psychiatry, reinforcing the importance of transdiagnostic assessment frameworks that capture shared and specific symptom dimensions across mental disorders.

Another central theme across this Research Topic concerns transdiagnostic psychological processes that contribute to mental health and psychopathology. Fedyk et al. evaluated a transdiagnostic clinical intervention combining mindfulness-based training with cognitive behavioral therapy for dissociation, demonstrating how targeting underlying psychological processes may improve outcomes across diagnostic categories. Vancappel et al., using topic modeling techniques applied to narratives from patients, relatives, clinicians, and the general population, identified common transdiagnostic distal factors and psychological processes, including emotional difficulties, relational problems, trauma, and self-related processes, that cut across diagnostic boundaries and contribute to heterogeneous presentations. In addition, Chen et al. showed that alexithymia mediates the relationship between negative life events and somatic symptoms, highlighting emotional processing deficits as a transdiagnostic mechanism linking stress and mental health symptoms. Collectively, these studies support the view that shared psychological and emotional regulation processes play a central role across mental health conditions, reinforcing transdiagnostic and process-oriented models of psychopathology, and informing more effective clinical interventions.

The study protocol by Orlandi et al. exemplifies a translational, biomarker-oriented perspective, proposing a longitudinal investigation of adolescents with suicidal ideation or suicidal behavior integrating psychological assessment with peripheral biomarkers related to inflammation, hypothalamic–pituitary–adrenal (HPA) axis activity, and blood–brain barrier integrity. The study is based on the hypothesis that suicidality is a multifactorial and transdiagnostic phenomenon resulting from the interaction between psychological vulnerability, stress-related neuroendocrine dysregulation, and neuroinflammatory processes. Importantly, this approach highlights the close interplay between biological systems and core psychological processes previously discussed, such as emotion regulation, stress responsivity, and cognitive control, reinforcing their integration within a multilevel framework. By combining biological and psychological measures, this study aims to identify biomarker-informed risk profiles and improve early detection and prevention strategies, illustrating the importance of translational and multilevel approaches in mental health research. In a complementary direction, Santamaría-Vázquez et al. investigated a multimodal non-pharmacological intervention combining respiratory biofeedback, neurofeedback, and median nerve stimulation in children with attention deficit hyperactivity disorder (ADHD). The study reported improvements in behavioral and attentional symptoms, accompanied by changes in EEG activity, particularly increased frontal theta power, suggesting that multimodal neuromodulation and biofeedback approaches may promote neuroplastic changes and modulate underlying neurocognitive and emotional regulatory processes. Together, these findings further support the integration of biomarker-based and psychological approaches within a unified, multilevel model of mental health.

In addition, evidence from a recent systematic review indicates that fibromyalgia and major depressive disorder share similar alterations in resting-state functional connectivity, particularly involving the insula, anterior cingulate cortex, and prefrontal regions, suggesting a common neurosignature underlying pain processing and emotional regulation (Tocchetto et al.). Furthermore, Mok et al., in a study on artificial intelligence in psychosis, demonstrated the potential of machine learning models to predict psychosocial functioning and clinical outcomes, highlighting the growing role of data-driven approaches in mental health research and personalized care. In a related perspective, Kashevarova et al. discussed the application of deep learning approaches in obsessive-compulsive disorder, emphasizing the importance of data-driven subtyping, integration of genetic and neuroimaging data, and explainable artificial intelligence to improve clinical translation and support precision psychiatry approaches. Together, these studies highlight the growing importance of integrating biomarkers, neurotechnology-based interventions, neuroimaging, and computational approaches to better understand the neurobiological mechanisms underlying mental and neurological disorders and to support the development of more personalized and translational clinical strategies.

Neelapu and Grant (2025) investigated clinical characteristics associated with natural recovery in trichotillomania and skin picking disorder, contributing to the transdiagnostic understanding of recovery processes. Their findings showed that individuals who naturally recovered did not present lower disorder severity compared to those with current symptoms, but they did show significantly lower rates of current psychiatric comorbidities, particularly depression and ADHD. Importantly, many individuals who recovered naturally continued to present subclinical symptoms or replaced the behavior with other repetitive behaviors, suggesting that recovery in body-focused repetitive behaviors may involve symptom substitution or partial remission rather than complete symptom resolution. These findings highlight the importance of comorbidities, emotional regulation processes, and behavioral mechanisms in recovery trajectories, supporting the view that mental health conditions should be understood dimensionally and longitudinally rather than as static diagnostic categories.

Another major theme emerging from this Research Topic concerns neurocognitive and brain–body mechanisms underlying mental health conditions. Carvalho proposed a neurocognitive framework in which executive dysfunction is conceptualized as a transdiagnostic mechanism underlying multiple forms of psychopathology, influencing cognitive control, emotion regulation, decision-making, and adaptive functioning. Importantly, these processes do not operate in isolation but as interrelated and dynamically interacting mechanisms, in which cognitive control, emotional regulation, and relational/contextual dimensions mutually influence one another. Within this framework, executive dysfunction may represent a core vulnerability embedded in this network of interactions, shaping how individuals regulate emotions, process information, and respond to environmental and interpersonal demands. This study reinforces the association between executive dysfunction and multiple forms of psychopathology, suggesting that it may represent a central mechanism within this interconnected system (Snyder et al., 2015; Zelazo and Cunningham, 2007). Other contributions examined interoception, emotion regulation, and neurocognitive mechanisms associated with emotional processing and mental health, further highlighting the importance of brain-body interactions as part of a coordinated system linking cognitive, emotional, and physiological processes across mental disorders (Khalsa et al., 2018).

Together, the studies included in this Research Topic reflect a broader conceptual transition in mental health research: a shift from disorder-based models toward dimensional, transdiagnostic, and mechanism-based frameworks. Across psychometric, clinical, neurocognitive, and biomarker studies, the collected works highlight the importance of shared psychological, cognitive, emotional, and biological processes that cut across traditional diagnostic boundaries. Importantly, these processes are not static but operate as dynamically interacting systems that evolve over time, shaped by continuous interactions between individuals and their internal and external environments. This body of work also illustrates an important conceptual progression in the field, moving from categorical diagnoses toward dimensional models, from dimensional models toward transdiagnostic mechanisms, and from mechanisms toward biomarker-informed and precision psychiatry approaches. Within this perspective, mental health conditions are better understood as emergent and temporally evolving patterns arising from the interplay of cognitive, emotional, behavioral, and biological processes across different timescales. These developments support the integration of dimensional assessment, neurocognitive mechanisms, brain-body processes, and biological markers within longitudinal and multilevel frameworks to better understand mental health conditions and to develop more precise and personalized interventions.

## Future directions

Future research in mental health should move toward integrative, multilevel, and longitudinal models that combine behavioral, cognitive, neurophysiological, and biological data. Advances in computational psychiatry, machine learning, digital phenotyping, wearable technologies, and neurotechnology may allow the identification of individual risk profiles, prediction of treatment response, and development of adaptive, precision-based and personalized interventions based on individual dimensional profiles rather than categorical diagnoses. Multimodal approaches integrating neuroimaging, electrophysiology, behavioral assessment, ecological momentary assessment, and biological markers may provide a more comprehensive understanding of mental health and mental disorders and support the identification of transdiagnostic protective factors buffering against psychopathology development. In this context, the future of mental health research and clinical practice will likely depend on the integration of dimensional psychopathology, transdiagnostic mechanisms, neurocognitive models, and biomarker-informed approaches within a precision psychiatry framework. This transition represents not only a methodological shift but also a conceptual shift in how mental disorders are understood: from categorical nosologic diagnoses to dynamic systems involving cognitive, emotional, behavioral, and biological processes interacting over time. Future research should continue to examine transdiagnostic mechanisms in longitudinal designs, testing their causal role in symptom development and whether mechanism-targeted interventions lead to greater improvements than symptom-based approaches.

## Conclusion

This Research Topic highlights the growing importance of transdiagnostic, dimensional, and translational approaches in mental health research. By focusing on underlying mechanisms rather than diagnostic categories, integrating cognitive, emotional, and biological processes, and developing mechanism-based interventions, this body of work represents an important step toward precision psychiatry and more personalized approaches to mental health care. Bridging fundamental neuroscience, clinical psychology, psychiatry, and translational research will be essential for improving prevention, diagnosis, and treatment of mental health conditions in the coming decades.

The articles included in this Research Topic can be broadly organized into three main domains: dimensional and transdiagnostic assessment and mechanisms, mechanism-based and transdiagnostic interventions, and translational and biomarker-oriented approaches, including neuroimaging and artificial intelligence. Together, these contributions reflect the ongoing transition in mental health research from disorder-based models toward dimensional, mechanism-based, and precision psychiatry frameworks.

The contributions converge toward a common conceptual perspective in which mental disorders are not viewed as isolated diagnostic categories but as complex and dynamic systems emerging from interactions between neurocognitive processes, emotional regulation mechanisms, environmental stressors, and biological vulnerability factors. From this perspective, transdiagnostic mechanisms such as executive dysfunction, emotion regulation difficulties, interoceptive processing, and stress-related neurobiological alterations may represent core dimensions underlying multiple forms of psychopathology and psychiatric presentations. Understanding these shared mechanisms through a transdiagnostic lens may support the development of more targeted, adaptive, and personalized interventions, contributing to a more integrative and mechanism-based model of mental health and mental disorders.

In this context, the paradigm shift from discrete diagnostic categories to transdiagnostic mechanisms represents not only a methodological change but also a conceptual transformation in how mental disorders are understood. Rather than discrete entities, mental health conditions may be better conceptualized as dynamic systems resulting from interactions between cognitive, emotional, behavioral, and biological processes over time. Future research may increasingly focus on identifying the mechanisms underlying symptom dimensions, the biological and cognitive systems involved, and the individual factors influencing risk, resilience, and treatment response. In this sense, transdiagnostic and multilevel approaches may represent one of the most promising paths toward a more integrative and precise science of mental health.
